# P39 A qualitative study investigating the views of nurses and doctors on the management of urinary tract infections in long-term care facilities

**DOI:** 10.1093/jacamr/dlad066.043

**Published:** 2023-06-14

**Authors:** Aoife Fleming, Áine Monaghan, Natasha Benjamin-Payne, Mala Shah, Teresa M Barbosa

**Affiliations:** Pharmaceutical Care Research Group, School of Pharmacy, University College Cork, Cork, Ireland; Department of Pharmacy, Mercy University Hospital, Cork, Ireland; Pharmaceutical Care Research Group, School of Pharmacy, University College Cork, Cork, Ireland; Pharmaceutical Care Research Group, School of Pharmacy, University College Cork, Cork, Ireland; Quality and Patient Safety, Health Service Executive, Cork, Ireland; Pharmaceutical Care Research Group, School of Pharmacy, University College Cork, Cork, Ireland

## Abstract

**Background:**

The most commonly reported infections in older persons’ long-term care facilities (LTCFs) are urinary tract infections (UTIs). UTIs contribute to high rates of antimicrobial prescribing, for the treatment and prophylaxis of UTI. Antimicrobials are often inappropriately prescribed and can increase the risk of antimicrobial resistance and other negative outcomes in LTCF populations.^1^

**Objectives:**

To explore the views and experiences of nurses and doctors working in LTCF on the management of UTIs.

**Methods:**

Qualitative, semi-structured interviews were conducted with nurses and GPs who work in or provide medical service in LTCFs in November 2022. Interviews were conducted online or by telephone, and recordings transcribed. Thematic analysis was conducted, and the themes were mapped to the Theoretical Domains Framework (TDF)1.^2^

**Results:**

Seven nurses and eight GPs were interviewed. Key domains such as knowledge, memory, attention and decision making and beliefs about consequences had an influential role on UTI management as reported by participants. Contextual factors such as the time taken to obtain urine culture results, and social factors such as the key role of the LTCF nurse, were reported as key issues. Variability in practice and inconsistent knowledge of urine dipstick and UTI management guidelines were acknowledged in the management of UTIs and urine dipstick practices between participants.

**Conclusions:**

This study identified social factors, contextual LTCF factors and variability in the management of UTI and urine dipstick practices across the participants. LTCFs require improved access to urine culture results, improved communication between all healthcare professionals involved and increased implementation of dipstick and UTI management guidelines.
